# Supplementary material for: Gender differences in trunk appearance perception and health-related quality of life (HRQoL) in the patients with moderate adolescent idiopathic scoliosis (AIS) undergoing orthotic treatment: An observational study
Source: PLoS One. 2025 Jun 25;20(6):e0325383. doi: 10.1371/journal.pone.0325383 (PMC12193679; doi:10.1371/journal.pone.0325383)

**Chinese version of Trunk Appearance Perception Scale (TAPS)**

身體外觀感知表

The TAPS include 3 sets of figures that depict the trunk from 3 viewpoints: each drawing is scored from 1 (greatest deformity) to 5 (smallest deformity) and a mean score is obtained by adding the scores for the 3 drawings and dividing by 3.

身體外觀感知表（TAPS）包括三組從三個角度描述身體外觀的圖片：每組圖片的評分是從1（變形程度最嚴重）到5（變形程度最輕），然後通過將三組圖片的得分相加並除以三以得出平均得分。


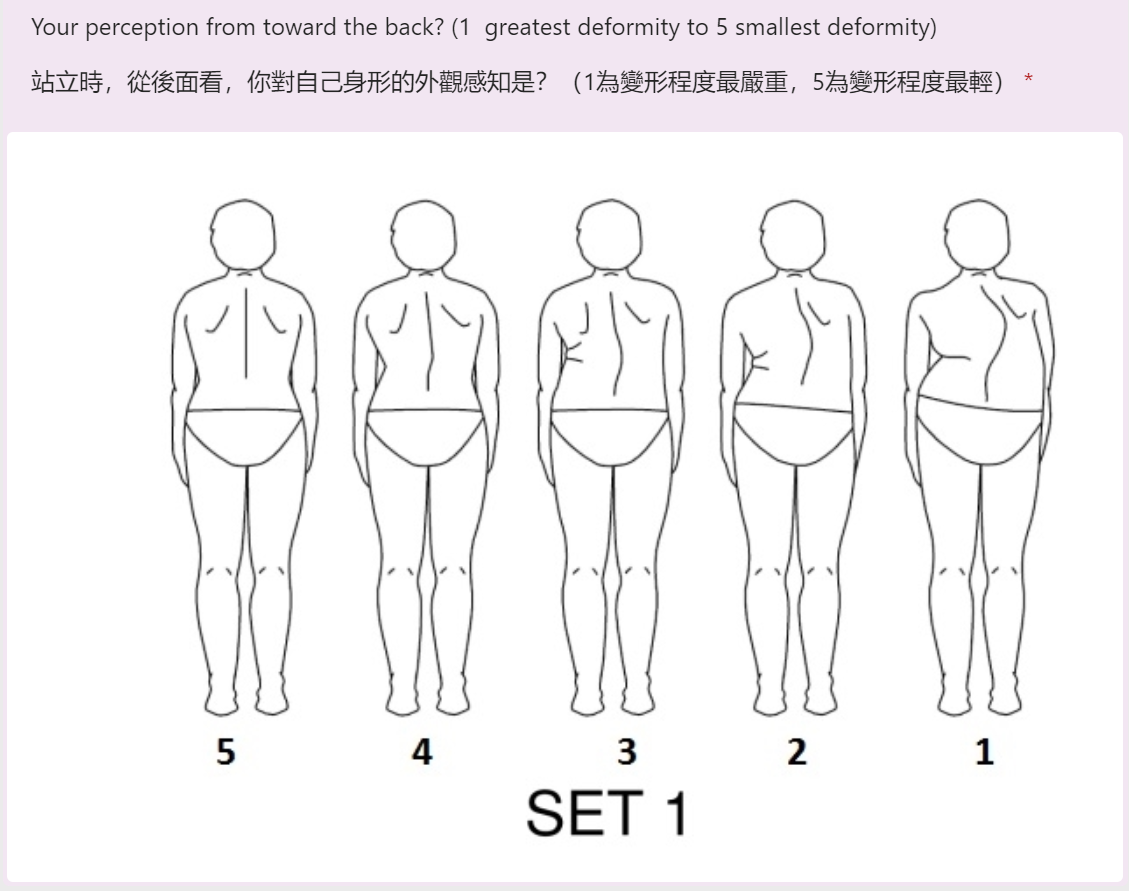


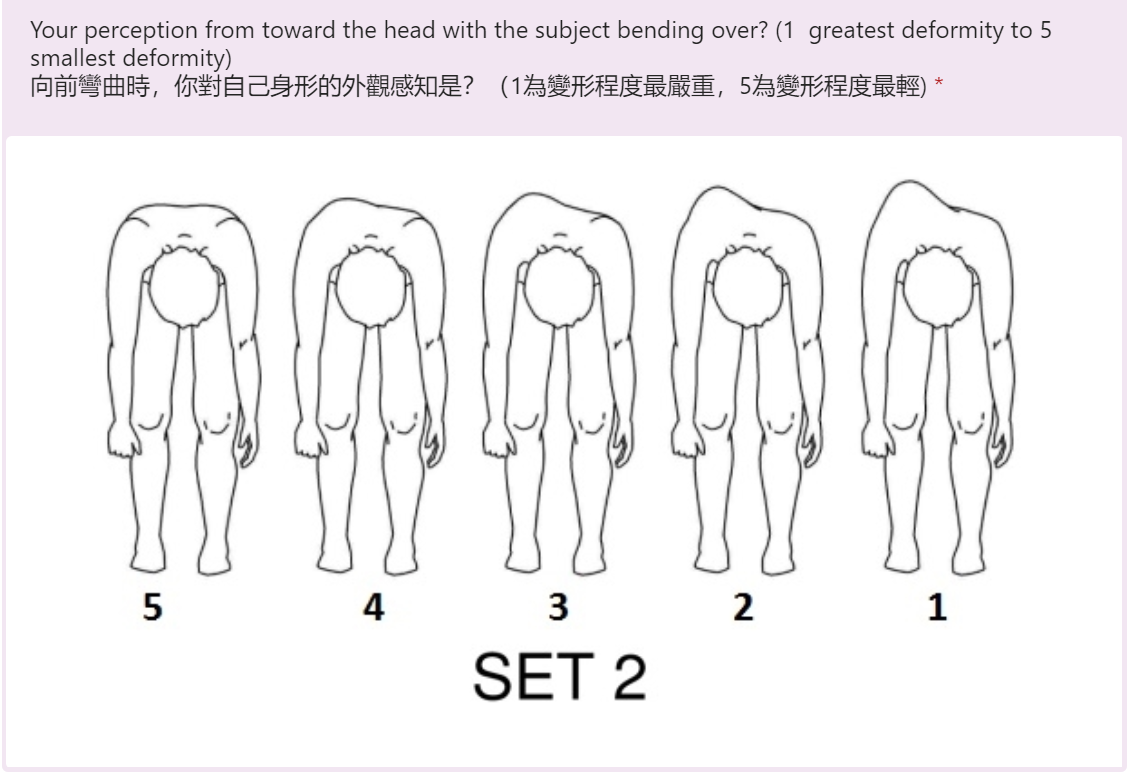


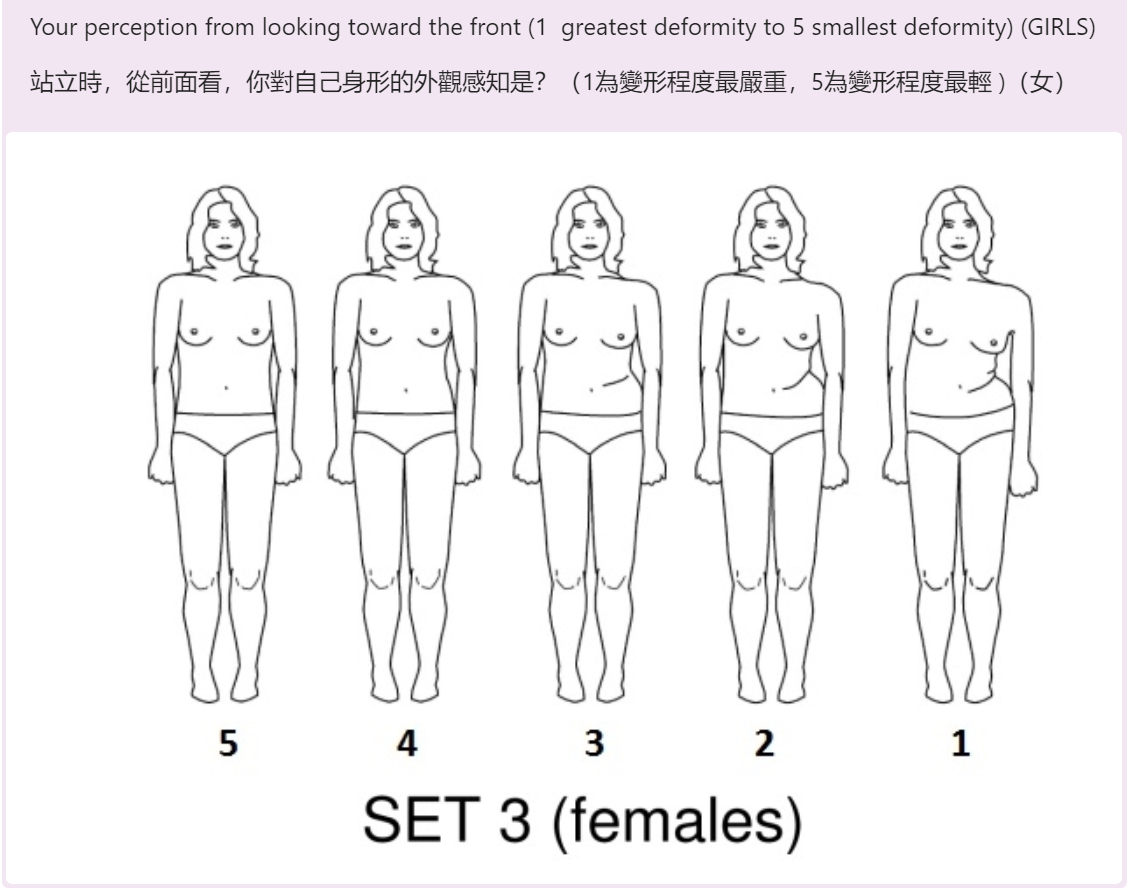


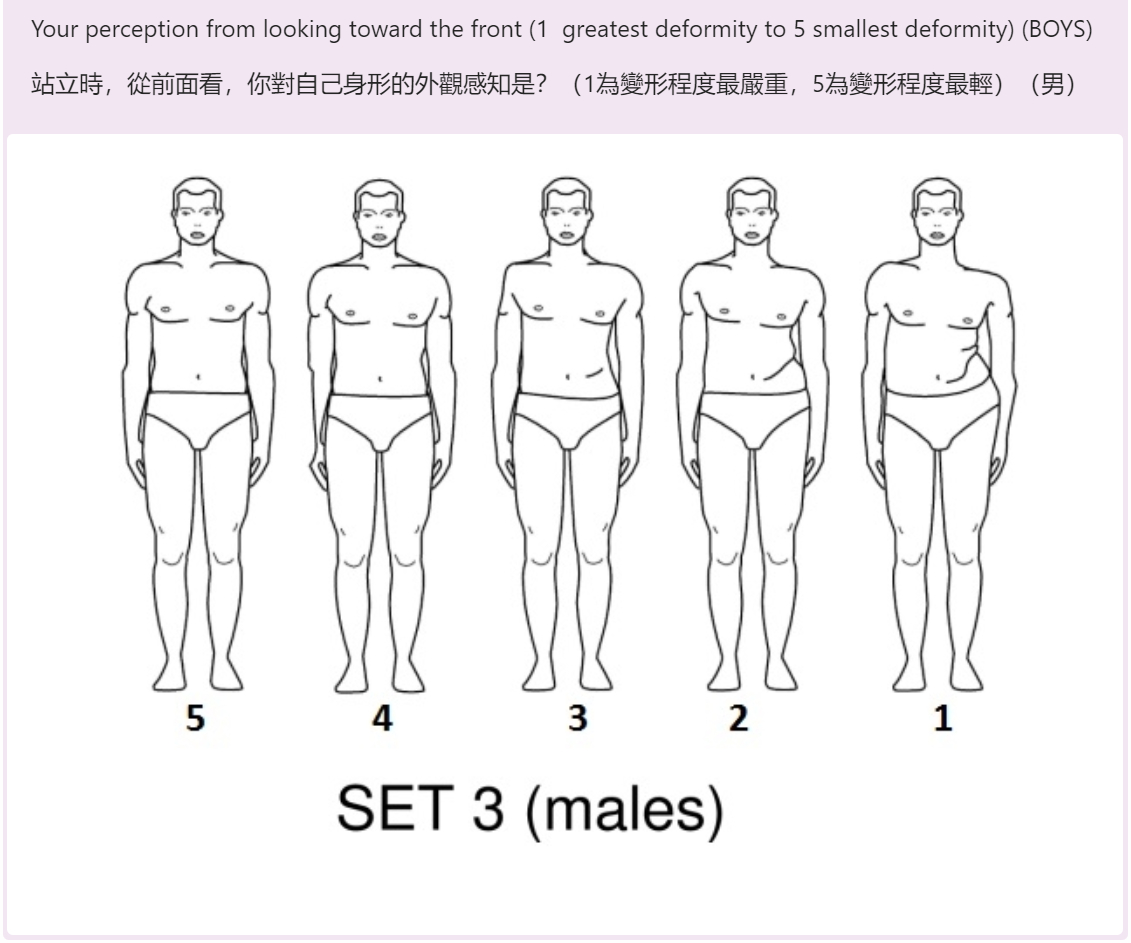

Supplement: S1 File — (DOCX) [file pone.0325383.s001.docx]
