# Supplementary material for: Gender differences in trunk appearance perception and health-related quality of life (HRQoL) in the patients with moderate adolescent idiopathic scoliosis (AIS) undergoing orthotic treatment: An observational study
Source: PLoS One. 2025 Jun 25;20(6):e0325383. doi: 10.1371/journal.pone.0325383 (PMC12193679; doi:10.1371/journal.pone.0325383)
Supplement: S2 File — (DOCX) [file pone.0325383.s002.docx]

**SRS-22r Patient Questionnaire**

**SRS-22病人問卷**

**INSTRUCTIONS:** We are carefully evaluating the condition of your back and it is **IMPORTANT THAT YOU ANSWER EACH OF THESE QUESTIONS YOURSELF.** Please **CIRCLE THE ONE BEST ANSWER TO EACH QUESTION.**

**指示:** 我們正在小心評估你背部的情況，因此**問卷上的每一條問題必須由你親自回答。** 請在每一條問題所提供的選擇中，**小心圈出你認為最正確的一個答案。**

1. Which one of the following best describes the amount of pain you have experienced during the past 6 months? 以下哪一項最能夠準確描述你在過去六個月所感受到痛楚的程度？

🞎 None 無痛楚

🞎 Mild 輕微

🞎 Moderate 中等

🞎 Moderate to severe中等至嚴重

🞎 Severe嚴重

2. Which one of the following best describes the amount of pain you have experienced over the last month? 以下哪一項最能夠準確描述你在過去一個月所感受到痛楚的程度？

🞎 None 無痛楚

🞎 Mild 輕微

🞎 Moderate 中等

🞎 Moderate to severe中等至嚴重

🞎 Severe嚴重

3. During the past 6 months have you been a very nervous person? 整體來說，在過去六個月期間你有感到十分焦慮嗎？

🞎 None of the time 完全沒有

🞎 A little of the time 小部份時間

🞎 Some of the time 有時

🞎 Most of the time大部份時間

🞎 All of the time 全部時間

4. If you had to spend the rest of your life with your back shape as it is right now, how would you feel about it? 如果你必須在背部維持現狀不變的情況下繼續生活，你會有甚麼感受？

🞎 Very happy 十分愉快

🞎 Somewhat happy 某程度上愉快

🞎 Neither happy nor unhappy 沒有愉快或不愉快

🞎 Somewhat unhappy 某程度上不愉快

🞎 Very unhappy 十分不愉快

5. What is your current level of activity? 你現時的活動能力如何？

🞎 Bedridden 只限於床上

🞎 Primarily no activity 基本上不能活動

🞎 Light labor and light sports 些微的運動及勞動

🞎 Moderate labor and moderate sports 有限度的運動及勞動

🞎 Full activities without restriction 活動不受限制

6. How do you look in clothes? 你在穿上衣服後的外觀如何？

🞎 Very good 很好

🞎 Good 好

🞎 Fair 可以接受

🞎 Bad 差勁

🞎 Very bad 十分差勁

7. In the past 6 months have you felt so down in the dumps that nothing could cheer you up? 在過去六個月期間你曾感到十分沮喪以至於任何事物也不能讓你開懷嗎？

🞎 Very often 經常

🞎 Often 大多數時間

🞎 Sometimes 有時

🞎 Rarely 很少數時間

🞎 Never 完全沒有

8. Do you experience back pain when at rest? 你在休息時背部有感到疼痛嗎？

🞎 Very often 經常

🞎 Often 大多數時間

🞎 Sometimes 有時

🞎 Rarely 很少數時間

🞎 Never 完全沒有

9. What is your current level of work/school activity? 你現時在工作/學校的活動能力為多少？

🞎 100% normal 正常的 100%

🞎 75% normal 正常的 75%

🞎 50% normal 正常的 50%

🞎 25% normal 正常的 25%

🞎 0% normal 正常的 0%

10. Which of the following best describes the appearance of your trunk; defined as the human body except for the head and extremities? 以下哪一項最能夠描述你軀幹的外觀？(軀幹的定義為人的身體除去頭部及四肢)

🞎 Very good 很好

🞎 Good 好

🞎 Fair 可以接受

🞎 Bad 差勁

🞎 Very bad 十分差勁

11. Which one of the following best describes your pain medication use for back pain? 下例哪一項最能準確地描述你因背部疼痛而所需要服用的藥物？

🞎 None 無

🞎 Non-narcotics weekly or less (e.g., aspirin, Tylenol, Ibuprofen) 一般止痛藥 (每星期服用一次或更少)

🞎 Non-narcotics daily 一般止痛藥 (天天服用)

🞎 Narcotics weekly or less (e.g. Tylenol III, Lorcet, Percocet) 特效止痛藥 (每星期服用一次或更少)

🞎 Narcotics daily 特效止痛藥 (天天服用)

12. Does your back limit your ability to do things around the house? 你的背部疼痛有否影響你做家務的能力？

🞎 Never 沒有

🞎 Rarely 少許

🞎 Sometimes 某程度上有

🞎 Often 很大程度上有

🞎 Very often 經常有

13. Have you felt calm and peaceful during the past 6 months? 整體來說，你在過去六個月期間有感到安寧和平靜嗎？

🞎 All of the time 經常

🞎 Most of the time大多數時間

🞎 Some of the time 有時

🞎 A little of the time 很少數時間

🞎 None of the time 完全沒有

14. Do you feel that your back condition affects your personal relationships? 你有否感到你背部的狀況對你的人際關係構成影響？

🞎 Never 沒有

🞎 Rarely 少許

🞎 Sometimes 某程度上有

🞎 Often 很大程度上有

🞎 Very often 經常有

15. Are you and/or your family experiencing financial difficulties because of your back? 你以及/或你的家人有否因為你背部的問題而在經濟方面遇到困難？

🞎 Severely 極有

🞎 Moderately 很大程度上有

🞎 Mildly 某程度上有

🞎 Slightly 少許

🞎 None 沒有

16. In the past 6 months have you felt down hearted and blue? 整體來說，在過去六個月期間你有否感到失落和灰心？

🞎 Never 完全沒有

🞎 Rarely 很少數時間

🞎 Sometimes 有時

🞎 Often 大多數時間

🞎 Very often 經常

17. In the last 3 months have you taken any days off of work, including household work, or school because of back pain? 在過去三個月期間你有否因背痛而向學校/公司請假？如有，共有多少天？

🞎 0 days 零天

🞎 1 day 一天

🞎 2 days 兩天

🞎 3 days 三天

🞎 4 or more days 四天或以上

18. Does your back condition limit your going out with friends/family? 你背部的狀況有否阻礙你和家人/朋友外出？

🞎 Never 從來沒有

🞎 Rarely 很少數時間

🞎 Sometimes 有時

🞎 Often 大多數時間

🞎 Very often 經常

19. Do you feel attractive with your current back condition? 你現時背部的狀況會否讓你覺得自己仍有吸引力？

🞎 Yes, very 會，很有吸引力

🞎 Yes, somewhat 會，某程度上有吸引力

🞎 Neither attractive nor unattractive 無影響

🞎 No, not very much 否，沒有甚麼吸引力

🞎 No, not at all 否，完全沒有吸引力

20. Have you been a happy person during the past 6 months? 整體來說，你在過去的六個月裏感到愉快嗎？

🞎 None of the time 完全沒有

🞎 A little of the time 很少數時間

🞎 Some of the time 有時

🞎 Most of the time大多數時間

🞎 All of the time 經常

21. Are you satisfied with the results of your back management? 你對你背部治療的成效感到滿意嗎？

🞎 Very satisfied 十分滿意

🞎 Satisfied 滿意

🞎 Neither satisfied nor unsatisfied 不是滿意也不是不滿意

🞎 Unsatisfied 不滿意

🞎 Very unsatisfied 非常不滿意

22. Would you have the same management again if you had the same condition? 如果你的背部再次遇到同類的情況你會否接受同樣的治理？

🞎 Definitely yes 一定會

🞎 Probably yes 可能會

🞎 Not sure 不清楚

🞎 Probably not 可能不會

🞎 Definitely not一定不會

Thank you for completing this questionnaire. Please comment if you wish. 多謝你的合作，如有任何意見請填寫在以下的空位上。

**Marks: higher scores representing greater patient quality of life.**
