# Supplementary material for: Gender differences in trunk appearance perception and health-related quality of life (HRQoL) in the patients with moderate adolescent idiopathic scoliosis (AIS) undergoing orthotic treatment: An observational study
Source: PLoS One. 2025 Jun 25;20(6):e0325383. doi: 10.1371/journal.pone.0325383 (PMC12193679; doi:10.1371/journal.pone.0325383)
Supplement: S3 File — (DOCX) [file pone.0325383.s003.docx]

**Brace Questionnaire (BrQ)**

**關於支架的問卷**

This questionnaire asks how you feel about your health, while you are wearing a brace. This is not a test and there are no right or wrong answers. 下面的問卷包含的問題有關你對你的健康的想法和感受，答復沒有對與錯。

• Please read carefully every question 請認真閱讀每個問題

• Choose the best answer and mark with an x 選擇一個你認為更適合你的答案，在旁邊的放框中打x


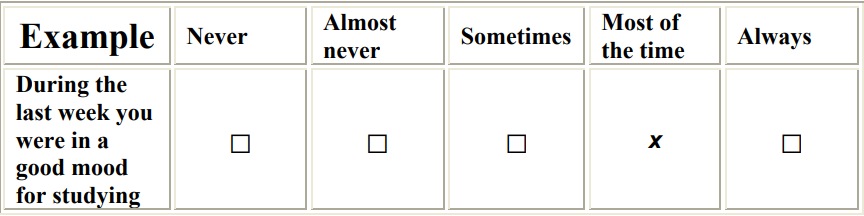


1. You are wearing the brace since (month/year). 你從 （月/年）開始配戴支架。

2. You are wearing the brace for hours/day.你每天配戴支架 小時。

| During the past 3 months…在過去的三個月中， | Never 從來沒有 | Almost never很少 | Sometimes 有時候 | Most of the time 經常 | Always 一直有 |
| --- | --- | --- | --- | --- | --- |
| 3. The brace made you feel ill.支架讓你感覺自己是病人 | 100🞎 | 80🞎 | 60🞎 | 40🞎 | 20🞎 |
| 4. You were afraid that your back will get worse.你擔心你的脊柱側彎會惡化。 | 100🞎 | 80🞎 | 60🞎 | 40🞎 | 20🞎 |

| During the past 3 months while you were wearing the brace…在過去的三個月中， | Never 從來沒有 | Almost never很少 | Sometimes 有時候 | Most of the time 經常 | Always 一直有 |
| --- | --- | --- | --- | --- | --- |
| 5. You felt tired when walking. 你因為戴著支架走路會累。 | 100🞎 | 80🞎 | 60🞎 | 40🞎 | 20🞎 |
| 6. You were able to run. 你可以戴著支架跑步 | 20🞎 | 40🞎 | 60🞎 | 80🞎 | 100🞎 |
| 7. You managed to wear the brace without any help. 你可以在沒有人幫助的情況下自己戴支架。 | 20🞎 | 40🞎 | 60🞎 | 80🞎 | 100🞎 |
| 8. You managed to take out the brace without any help. 你可以在沒有人幫助的情況下自己脫下支架。 | 20🞎 | 40🞎 | 60🞎 | 80🞎 | 100🞎 |
| 9. You couldn’t eat well. 你因為戴著支架而吃不好。 | 100🞎 | 80🞎 | 60🞎 | 40🞎 | 20🞎 |
| 10. You couldn’t sleep well. 你因為戴著支架而睡不好。 | 100🞎 | 80🞎 | 60🞎 | 40🞎 | 20🞎 |
| 11. You couldn’t breath well. 你因為戴著支架而不能呼吸。 | 100🞎 | 80🞎 | 60🞎 | 40🞎 | 20🞎 |

| During the past 3 months…在過去的三個月中， | Never 從來沒有 | Almost never很少 | Sometimes 有時候 | Most of the time 經常 | Always 一直有 |
| --- | --- | --- | --- | --- | --- |
| 12. The brace made you feel nervous. 支架讓你感到煩躁。 | 100🞎 | 80🞎 | 60🞎 | 40🞎 | 20🞎 |
| 13. You felt worried because of the brace. 因為支架而感到傷心。 | 100🞎 | 80🞎 | 60🞎 | 40🞎 | 20🞎 |
| 14. You felt happy. 感到幸福。 | 20🞎 | 40🞎 | 60🞎 | 80🞎 | 100🞎 |
| 15. You believed that your life would be better if you were not on brace. 你相信如果不用配戴支架的話，你會生活得更好。 | 100🞎 | 80🞎 | 60🞎 | 40🞎 | 20🞎 |
| 16. You believed that brace treatment was beneficial. 你認為支架療法對你有用。 | 20🞎 | 40🞎 | 60🞎 | 80🞎 | 100🞎 |

| During the past 1 month…在過去的一個月中， | Never 從來沒有 | Almost never很少 | Sometimes 有時候 | Most of the time 經常 | Always 一直有 |
| --- | --- | --- | --- | --- | --- |
| 17. You felt proud of yourself. 你對自己感到驕傲。 | 20🞎 | 40🞎 | 60🞎 | 80🞎 | 100🞎 |
| 18. You were satisfied with your body. 對自己感到滿意。 | 20🞎 | 40🞎 | 60🞎 | 80🞎 | 100🞎 |

| During the past 1 month…在過去的一個月中， | Never 從來沒有 | Almost never很少 | Sometimes 有時候 | Most of the time 經常 | Always 一直有 |
| --- | --- | --- | --- | --- | --- |
| 19. You felt strong and full of energy. 你感覺很有勁和精力充沛。 | 20🞎 | 40🞎 | 60🞎 | 80🞎 | 100🞎 |
| 20. You felt tired and exhausted because of the brace. 你因為戴著支架感覺疲憊和精疲力盡。 | 100🞎 | 80🞎 | 60🞎 | 40🞎 | 20🞎 |

| During the past 1 month, because of the brace…在過去的一個月中，因為支架， | Never 從來沒有 | Almost never很少 | Sometimes 有時候 | Most of the time 經常 | Always 一直有 |
| --- | --- | --- | --- | --- | --- |
| 21. You had difficulties with your lessons.你上課有困難。 | 100🞎 | 80🞎 | 60🞎 | 40🞎 | 20🞎 |
| 22. You were absent from school.你曠課了。 | 100🞎 | 80🞎 | 60🞎 | 40🞎 | 20🞎 |
| 23. You found it hard to pay attention in the classroom. 在課堂上走神。 | 100🞎 | 80🞎 | 60🞎 | 40🞎 | 20🞎 |

| During the past 1 month, while you were wearing the brace…在過去的一個月中，當你穿著支架， | Never 從來沒有 | Almost never很少 | Sometimes 有時候 | Most of the time 經常 | Always 一直有 |
| --- | --- | --- | --- | --- | --- |
| 24. You had to take medication for pain. 因為痛吃藥了。 | 100🞎 | 80🞎 | 60🞎 | 40🞎 | 20🞎 |
| 25. You had pain during the night. 夜裡痛。 | 100🞎 | 80🞎 | 60🞎 | 40🞎 | 20🞎 |
| 26. You had pain when walking. 走路時痛。 | 100🞎 | 80🞎 | 60🞎 | 40🞎 | 20🞎 |
| 27. You had pain when sitting. 坐著時痛。 | 100🞎 | 80🞎 | 60🞎 | 40🞎 | 20🞎 |
| 28. You had pain when climbing stairs. 上下樓梯時痛。 | 100🞎 | 80🞎 | 60🞎 | 40🞎 | 20🞎 |
| 29. You felt pins and needles to your arms or legs. 因戴支架手腳麻。 | 100🞎 | 80🞎 | 60🞎 | 40🞎 | 20🞎 |

| During the past 1 month, because of the brace…在過去的一個月中， | Never 從來沒有 | Almost never很少 | Sometimes 有時候 | Most of the time 經常 | Always 一直有 |
| --- | --- | --- | --- | --- | --- |
| 30. You couldn’t go out with your friend. 支架影響你與朋友在一起。 | 100🞎 | 80🞎 | 60🞎 | 40🞎 | 20🞎 |
| 31. Your friends felt compassion for you. 你的朋友因為你的背脊問題而憐憫你。 | 100🞎 | 80🞎 | 60🞎 | 40🞎 | 20🞎 |
| 32. You felt different from your peers. 因為戴支架你感覺與朋友們不一樣。 | 100🞎 | 80🞎 | 60🞎 | 40🞎 | 20🞎 |
| 33. You had problems with your family.因為支架你與你家人有些問題。 | 100🞎 | 80🞎 | 60🞎 | 40🞎 | 20🞎 |
| 34. You believed that your relationship with your family or your friends would be better if you were not on brace. 你認為如果沒有支架你與你家人和朋友的關係會更好一些。 | 100🞎 | 80🞎 | 60🞎 | 40🞎 | 20🞎 |
| 1035. You stayed at home because you were ashamed. 因戴支架而羞於出門。 | 100🞎 | 80🞎 | 60🞎 | 40🞎 | 20🞎 |
| 36. You worn special clothes. 因為支架而穿特殊的衣服。 | 100🞎 | 80🞎 | 60🞎 | 40🞎 | 20🞎 |
