# Supplementary material for: Gender differences in trunk appearance perception and health-related quality of life (HRQoL) in the patients with moderate adolescent idiopathic scoliosis (AIS) undergoing orthotic treatment: An observational study
Source: PLoS One. 2025 Jun 25;20(6):e0325383. doi: 10.1371/journal.pone.0325383 (PMC12193679; doi:10.1371/journal.pone.0325383)
Supplement: Attachment — Submitted filename: Response to Reviewers.docx [file pone.0325383.s005.docx]

**Dear Sir/Madam,**

Thank you for the opportunity to revise this manuscript and for the constructive comments provided by the reviewers. We appreciate the time and effort invested in evaluating this work. Below, please find our point-by-point responses and the corresponding changes made in the revised manuscript.

**Comments to the Author**

1. Is the manuscript technically sound, and do the data support the conclusions?

The manuscript must describe a technically sound piece of scientific research with data that supports the conclusions. Experiments must have been conducted rigorously, with appropriate controls, replication, and sample sizes. The conclusions must be drawn appropriately based on the data presented.

Reviewer #1: Partly

Reviewer #2: Partly

2. Has the statistical analysis been performed appropriately and rigorously?

Reviewer #1: Yes

Reviewer #2: Yes

3. Have the authors made all data underlying the findings in their manuscript fully available?

The [PLOS Data policy](http://track.editorialmanager.com/CL0/http:%2F%2Fwww.plosone.org%2Fstatic%2Fpolicies.action%23sharing/1/010f01942ec1b508-71142361-dd96-4c73-a5a7-8e09e8c54d8f-000000/k3hIZqfjti2PNugmAOajjqOamcUj3BFYAa-nA4NFsbo=192) requires authors to make all data underlying the findings described in their manuscript fully available without restriction, with rare exception (please refer to the Data Availability Statement in the manuscript PDF file). The data should be provided as part of the manuscript or its supporting information, or deposited to a public repository. For example, in addition to summary statistics, the data points behind means, medians and variance measures should be available. If there are restrictions on publicly sharing data—e.g. participant privacy or use of data from a third party—those must be specified.

Reviewer #1: Yes

Reviewer #2: Yes

4. Is the manuscript presented in an intelligible fashion and written in standard English?

PLOS ONE does not copyedit accepted manuscripts, so the language in submitted articles must be clear, correct, and unambiguous. Any typographical or grammatical errors should be corrected at revision, so please note any specific errors here.

Reviewer #1: Yes

Reviewer #2: Yes

5. Review Comments to the Author

Please use the space provided to explain your answers to the questions above. You may also include additional comments for the author, including concerns about dual publication, research ethics, or publication ethics. (Please upload your review as an attachment if it exceeds 20,000 characters)

**Reviewer #1:** The study addresses an important topic, and the effort put forth by the authors in exploring the relationship between bracing and quality of life is commendable. However, several aspects of the manuscript require clarification and improvement to enhance its quality and readability.

Abstract:
**Comment:** Lines 5–9: The sentences lack logical flow, which hinders readability. Additionally, the rationale for the research is not adequately justified. Please provide a clearer explanation of the problem being addressed and the study’s significance.

**Response:** Thank you for your suggestions. We have revised these lines to clearly explain the problem being addressed and to emphasize the study’s significance. In the abstract section, lines 3-8:

“Although prior studies have evaluated various factors influencing quality of life (QoL) in the patients with AIS, the association between trunk appearance perception and QoL, including potential gender differences, remains insufficiently defined. This study investigated gender differences in trunk appearance perception and its relationship with QoL among the patients with moderate AIS undergoing orthotic treatment.”

**Comment:** Regarding the TAPS scale: Is it considered a subscale of quality of life (QoL)? If so, the title is misleading as it separates TAPS and QoL into distinct parameters.

**Response:** Thank you for your comments. TAPS is specifically designed to assess the subjective perception of trunk appearance, which is a feeling of body shape/image. Although body image and self-perception can influence psychological well-being and overall QoL, TAPS is not a subscale of any multifaceted QoL instrument such as the SRS-22r. Instead, it represents an independent measure within the broader context of psychosocial factors that may relate to QoL. To avoid any potential ambiguity, we modified the title so that it clearly indicates that trunk appearance perception (as measured by TAPS) and QoL are independent parameters examined for their association rather than any components of one another:

“Gender differences in trunk appearance perception and its association with quality of life in the patients with moderate adolescent idiopathic scoliosis undergoing orthotic treatment: An observational study”

Clarifications were also added in the introduction section, lines 52-60:

“QoL is a multidimensional concept that includes physical health, functional ability, emotional well-being, and social relationships [1-3]. In the patients with moderate AIS, both spinal deformity and long-term orthotic treatment can lead to psychosocial challenges [4-6]. In particular, trunk appearance perception, how people subjectively evaluate the aesthetic qualities of their torso, plays a key role in overall body image. Changes in trunk appearance can affect self-esteem and emotional health, thereby influencing social interactions and overall QoL [7]. Moreover, prolonged orthosis use can alter lifestyle and increase stress, anxiety, and depression, further reducing self-esteem and leading to a decline in overall QoL [8-10].”

Introduction and Methods:

**Comment:** Lines 40–44: The claims made here require appropriate references to support them.

**Response:** References have been added to address the comment in the introduction section, lines 46-49:

“However, the success of orthotic treatment depends not only on its mechanical effectiveness but also on patient compliance, which can be challenging due to the physical discomfort, altered body appearance, and restrictions on daily activities imposed by orthosis wear [11, 12].”

**Comment:** Lines 55–60: There is a contradiction in this section. While you mention that QoL has been evaluated in both genders, you also state that further investigation is needed for males. What specific limitations in previous studies motivated this research? Please elaborate.

**Response:** Thank you for highlighting the contradiction and requesting further clarification. We have revised the text to explicitly state the limitations in the previous studies and clarify the motivation for our research, in the introduction section, lines 63-74:

“Current research highlights gender differences in these domains. Females with AIS tend to be more sensitive to their physical appearance and are more likely to experience body dissatisfaction, which is associated with lower QoL, particularly in areas related to body image and social functioning [4, 13]. Studies comparing overall body image perception and QoL between genders have produced mixed results: some have found no significant differences in stress levels, perception of spinal appearance, and QoL [6, 14], while others report that females exhibit lower body image perception and QoL during orthotic treatment [4, 13]. Moreover, there is limited research directly examining the relationship between trunk appearance perception and QoL in males versus females with AIS undergoing orthotic treatment. Although males also face appearance-related challenges, the nature and impact of these issues on their QoL may differ and require further analysis.”

**Comment:** Were all patients prescribed the same type of brace, or were there differences? If variations existed, clarify these details and discuss their potential impact.

**Response:** The same type of brace was prescribed for the patients. We revised the description for clarification, in the materials and methods section, lines 93-97:

**“**Each patient was prescribed a custom-fabricated Hong Kong style TLSO (Figure 1) by a qualified orthotist and instructed to wear it 23 hours a day. The orthosis was equipped with strap markings to ensure correct tightness. All patients received the same type of orthosis, thereby standardizing the treatment and minimizing potential variations in outcomes attributable to differences in orthosis design.”

**Comment:** Please include an image of the brace used in this study for context. Additionally, the current manuscript contains low-quality images. Replace these with high-resolution photos to ensure clarity and readability.

**Response:** Thank you for your suggestion. We have now included an image of the brace used in the study and replaced the low-resolution images with high-quality photos throughout the manuscript.

**Comment:** Lines 103–106: Initially, the third evaluation was described as being conducted 7 months after the start of bracing. Later, this timeline was adjusted due to the pandemic. Ensure consistency in reporting evaluation timelines to avoid confusion.

**Response:** The sentence was revised for clarification, in the materials and methods section, lines 123-130:

**“**The third evaluation was originally scheduled for six months after the second assessment (i.e., seven months post-orthosis fitting) to capture longer-term treatment effects [15]. An investigator provided explanations or clarifications as necessary. By the third evaluation, the patients were informed of their in-brace correction results. However, because of disruptions caused by the COVID-19 pandemic, some follow-up sessions were rescheduled to ensure participant safety and data collection, resulting in the third evaluation being completed between 6 and 17 months after having orthotic treatment, with an average follow-up period of 8.9 months.”

Results:

**Comment:** In the questionnaire results section, organize the data in a table that includes significance values to improve readability and facilitate interpretation of the findings.

**Response:** Thank you for your suggestion, the p-values were added in all results.

Discussion:

**Comment:** Lines 218–219: You discuss a finding that “for females, higher QoL was linked to an improved self-image,” but there is no corresponding result in the manuscript to confirm this claim. Ensure that all discussed findings are supported by results presented in the study.

**Response:** Thank you for your valuable comment. We have revised the discussion by rephrasing the statement to accurately reflect our findings.

**Comment:** Throughout the manuscript, some findings are either inconsistently reported or not adequately supported by the results section. For example:

You report no significant difference in TAPS scores between males and females across all evaluations, yet the discussion suggests otherwise. The second evaluation reportedly showed higher TAPS scores compared to the first evaluation, but this is misinterpreted in the discussion as males having better scores than females. This inconsistency must be corrected to ensure the discussion aligns with the reported results.

Ensure that all claims made in the discussion are clearly presented in the results section, complete with statistical significance values.

**Response:** Thank you for your valuable comment. We have carefully reexamined the discussion to ensure that every interpretation is supported by our results. Based on your feedback, we have revised the discussion section to directly reflect our data without extrapolating beyond our findings.

**Reviewer #2:** The author’s approach to exploring the correlation between gender and the psychological impact of adolescent idiopathic scoliosis (AIS) in adolescents who have undergone orthotic treatment is a smart and valuable choice. Overall, the manuscript is well-written and effectively highlights how scoliosis can affect the quality of life (QoL) differently across genders. The study used three well-known and validated questionnaires (TAPS, SRS22-r, and BrQ), completed by 39 participants (29 females, 10 males) at three time points: before starting treatment, one month after, and six months after the second visit. By applying appropriate statistical tests, the authors measured correlations between psychological factors and demographic features in each gender.

The findings were noteworthy: Though both genders had issues addressing self-esteem and poor self-image, each showed different sensitivity in each studied sub-domain. Remarkably, the QoL and trunk appearance perception showed a significant correlation in females, unlike males who showed more sensitivity to brace compliance and the duration of treatment.
The manuscript’s structure and clear articulation of research questions are commendable. However, there are a few areas where addressing certain shortcomings could significantly enhance the overall impact of the study.

Introduction
**Comment:** The introduction provides a concise overview of how gender differences can influence psychological parameters. However, it primarily focuses on the notion that females are more sensitive to these issues—a perspective often cited but potentially biased. While this viewpoint is supported by some studies, there is also evidence suggesting that males may be equally or more affected in certain scenarios. To ensure a balanced discussion, it would be beneficial to reference research supporting both perspectives.

**Response:** Thank you for your thoughtful comment regarding the potential bias in our introduction. We agree that while many studies highlight that females with AIS tend to exhibit greater sensitivity to body image issues, there are also some studies suggesting that no significant difference was found between males and females. In response, we have revised our introduction to incorporate a more balanced discussion.

Methodology
**Comment:** The timeline for administering the questionnaires—baseline, one month, and seven months—seems logical but would be more robust if supported by references justifying these specific intervals.

**Response:** Relevant references were added to address this comment, in the materials and methods section, lines 117-125:

**“**The evaluation timeline was based on clinical practice guidelines and some previous studies. The first evaluation, conducted before orthosis fitting, employed only the Trunk Appearance Perception Scale (TAPS) to capture baseline trunk appearance perception. One month after the start of orthotic treatment, an integrated questionnaire, comprising TAPS, the Scoliosis Research Society-22r (SRS-22r), and the Brace questionnaire (BrQ), was administered to assess early adaptation, allowing patients to adjust their lifestyle to the orthosis [16]. The third evaluation was originally scheduled for six months after the second assessment (i.e., seven months post-orthosis fitting) to capture longer-term treatment effects [15].”

**Comment:** Additionally, since the study utilized both English and Chinese versions of the questionnaires, it is important to address the reliability and validity of the Chinese versions in the text, along with relevant supporting data. The rationale for using English versions when the Chinese versions may be more culturally appropriate should also be clarified.

**Response:** Relevant references were added to support the use of Chinese-translated questionnaires, in the materials and methods section:

Lines 146-148:

“Similarly, the Chinese-translated TAPS has been validated in prior studies, showing acceptable internal consistency (Cronbach’s α = 0.70) and reproducibility (ICC = 0.95) [17].”

Lines 158-159:

“Moreover, the Chinese-translated SRS-22 questionnaire has shown excellent test-retest reproducibility, with an ICC of 0.75 across all domains [18].”

Lines 169-172:

“The original BrQ demonstrates satisfactory internal consistency reliability (Cronbach's alpha = 0.82) [19] and the Chinese-translated version has shown very good internal consistency (Cronbach's alpha = 0.89) and excellent test-retest reproducibility (ICC = 0.83)[20].”

In this study, we used both the English and Chinese versions of the questionnaires to accommodate the bilingual nature in Hong Kong. This dual-language approach enables direct comparisons with international studies while allowing participants to select their preferred language, thereby enhancing comprehension and data quality. Revision was added in the manuscript to address the comment, in the materials and methods section, lines 132-135:

“Three questionnaires were used in this study, with both English and Chinese versions available for patients in a bilingual society. This dual-language approach facilitates direct comparisons with international studies and ensures that participants can select their preferred language, enhancing comprehension and data quality.”

**Comment:** The choice to use both the SRS22-r and BrQ questionnaires is somewhat unclear. While BrQ is specifically designed for assessing brace treatment, SRS22-r is more commonly used for surgical cases and is known to have a ceiling effect in certain sub-domains like satisfaction and pain. Clarifying why SRS22-r was used alongside BrQ would help readers understand its inclusion. Additionally, while the BrQ is a suitable choice, the scoring methodology should be described in more detail.

**Response:** We used the BrQ because it is designed specifically to assess the patient experience during orthotic management, while the SRS22-r is widely used in scoliosis management, allowing us to compare our results with previous studies. Despite its ceiling effects in some areas, the SRS22-r provides general QoL aspects that complement the information from the BrQ, in the materials and methods section, lines 150-153:

“The SRS-22r questionnaire, recommended by the Scoliosis Research Society (SRS) for evaluating treatment outcomes, assesses health-related QoL in the patients with AIS and has been widely used in previous research as a comprehensive measure of overall QoL [4, 21-23].”

The scoring methodology was further explained in the manuscript, lines 163-168:

**“**Each item is rated on a 5-point Likert scale from 1 (poorest) to 5 (best). The total BrQ score is obtained by summing all 34 item scores, multiplying the sum by 20, and dividing by 34, yielding a normalized score between 20 and 100 (with higher scores indicating better QoL). A subscale score for each domain is calculated by dividing the domain’s total score by the number of items in that domain.”

**Comment:** The TAPS questionnaire also raises some concerns. The first and second sets of questions, respectively depicting the body from a back view and in a forward-bending position, are complicated to be answered subjectively by the patients. Alternatively, the Spinal Appearance Questionnaire (SAQ) could be an easier and more comprehensive option.

**Response:** We would like to clarify our rationale for using the TAPS in the study:

1. The back view and forward-bending view in the TAPS are specifically designed to capture how patients believe others perceive their trunk appearance, which is a critical component of self-image assessment in scoliosis.
2. The SAQ also includes the back view, forward-bending, and even backward-bending postures, comprising a total of 33 items. In this study, patients were required to complete additional questionnaires (SRS-22r and BrQ) to assess their QoL. Consequently, the extended length of the SAQ may contribute to respondent fatigue and redundancy, as some items overlap with those in the SRS-22r. For example, items assessing attractiveness or overall self-image appear in both tools, thereby reducing the efficiency of the assessment process.
3. The study population includes younger patients (ages 10–15) for whom some of the more abstract terms in the SAQ (e.g., “head chest hips” and “shoulder blade rotation”) can be difficult to understand, even when supported by illustrations, as reported in a previous study (Mulcahey et al., 2011). In contrast, the TAPS features only three straightforward items, which helps maintain clarity and minimizes cognitive load.
4. The SAQ was originally designed to capture both patients’ and their parents’ perceptions of spinal deformity. Since this study focuses on patients’ subjective evaluations of their own appearance, the TAPS provides a more targeted and practical assessment.

To further address the comment, we have revised the method section, lines 139-142:

“The inclusion of both the back view and the forward-bending view is for capturing how patients believe others perceive their trunk appearance, a key aspect of self-image in scoliosis [24].”

**Comment:** Another methodological issue is the decision to have participants complete all three questionnaires at the second and third time points, but only the TAPS at baseline. While it is understandable that BrQ could not be completed before brace treatment, the absence of SRS22-r at baseline is less clear. QoL at the start of treatment could significantly influence later outcomes, and the rationale for excluding this data should be explained.

**Response:** Thank you for raising this important point. SRS-22r includes a domain on treatment satisfaction, which might be less relevant when conducted before treatment. In this study, patients were asked to complete the full SRS-22r questionnaire after a short period of brace treatment. This one-month adaptation period allowed patients to have an initial treatment experience, thereby providing a more meaningful baseline evaluation of their QoL. By doing so, we aimed to capture early changes in QoL that are pertinent to the treatment experience, which can be a baseline result to compare to the QoL 6 months later. Some studies assessed the QoL before treatment by excluding the treatment-specific items in SRS-22r, which could provide additional insights into pre-treatment QoL related to the remaining domains. Future research may consider incorporating this modified approach to better understand the baseline status and its influence on later outcomes.

**Comment:** The inclusion criterion of a minimum 8-hour brace compliance also raises questions. Previous studies, such as Weinstein et al., suggest that the minimum compliance for a TLSO brace is 12.9 hours. Without direct literature supporting the 8-hour criterion, its use in this study is questionable.

**Response:** Thank you for your comments. Patients with high compliance tend to demonstrate greater acceptance of the orthosis, and including only these patients could bias our quality of life outcomes. To capture a realistic range of treatment behaviors and examine the dose-response relationship between brace wear time and QoL, we did not restrict daily compliance in our inclusion criteria. We observed one patient who wore the orthosis for only about 2 hours per day, likely providing insufficient exposure to the treatment, and consequently excluded this case (Sanders et al. 2014). In our study, average compliance ranged from 12.3 to 15.6 hours per day. We further revised the manuscript to clarify, in the Materials and methods section,

Lines 85-87:

“Eligibility criteria included: 1) age between 10 and 15 years; 2) moderate AIS with a major Cobb angle of 20 to 45 degrees; and 3) prescribed orthotic treatment.”

lines 99-102:

“However, two participants withdrew after one month due to discomfort from the orthosis, and one participant only wore the orthosis for around 2 hours per day, which may not have provided sufficient exposure to the orthotic treatment [25].”

**Comment:** Finally, while the authors mention using in-brace X-rays to calculate correction, the exact timing of these images should be specified.

**Response:** In our study, the in-brace X-rays were taken one month after the initial orthosis fitting. This timing was chosen to allow patients sufficient time to adapt to the orthosis, ensuring that the measured correction accurately reflects the orthosis’s effect on spinal alignment. We have updated the Methods section to clearly specify this timing, lines 108-113:

“To evaluate the corrective effect of the TLSO, the in-brace X-ray was obtained one month after the initial orthosis fitting. This timing was selected to allow patients sufficient time to adapt to the orthosis. Furthermore, the patients were required to wear the brace continuously for a full day before imaging to ensure that the measured correction accurately reflected its maximal effect on spinal deformities [26].”

Results
**Comment:** The statistical analysis appears robust and well-executed. The data is presented clearly, and the small sample size is appropriately acknowledged as a limitation. However, providing a preliminary sample size calculation to justify the initial selection of 42 participants would add credibility to the study.

**Response:** We conducted a power analysis for the Mann–Whitney U test (α = 0.05, 80% power, and a superiority probability of 0.68), which indicated a need for 42 participants. Due to recruitment challenges, we enrolled 39 (29 females and 10 males), reflecting the natural AIS prevalence (female-to-male ratio ≈ 10:1). While the smaller male subgroup may slightly reduce power for detecting gender differences, the use of a non-parametric test (the Mann–Whitney U test) helps mitigate this issue. We have also added the information in the method section, lines 97-100:

“A pre-study power analysis for the Mann–Whitney U test (α = 0.05, 80% power, and a superiority probability of 0.68 based on clinical and literature considerations [27]) indicated that 42 participants were required.”

**Comment:** In addition, including p-values in all tables would enhance transparency. For example, the absence of p-values in Table 2 could lead to some ambiguity.

**Response:** Thank you for your suggestion, the p-values were added in all tables.

**Comment:** The discussion of limitations is thorough, but one additional factor could be considered: the uneven follow-up periods caused by the COVID-19 pandemic. This might have influenced the results and should be noted.

**Response:** An additional limitation was added in the discussion section to address the comment, in lines 359-361:

“Furthermore, the uneven follow-up periods caused by the COVID-19 pandemic might have introduced additional variability in the results, affecting the generalizability of the findings.”

Discussion
**Comment:** While the discussion addresses an interesting topic, it lacks sufficient supporting evidence and would benefit from a major revision.

For example, the manuscript mentions societal pressures on young females regarding appearance but does not fully explore the topic: considering the sensitive age of adolescence and cultural differences in each society, not only females but also males could be prone to such difficulties. Although these pressures undeniably exist, the statement would be stronger with more supporting evidence. Exploring the role of cultural factors alongside societal pressures could add depth to the discussion.

**Response:** Thank you for your valuable comment. We have carefully reexamined the discussion to ensure that every interpretation is firmly supported by our results. Based on your feedback, we have revised the discussion section to directly reflect our data without extrapolating beyond our findings.

**Comment:** Similarly, the statement that males are more sensitive to the discomfort caused by braces is interesting but unsupported by measured data, as discomfort was not explicitly included as a parameter in the study.

**Response:** Thank you for your insightful comment. We have revised the discussion section to remove the unsupported claim regarding males being more sensitive to brace discomfort.

**Comment:** The discussion also notes that males with more severe scoliosis are more aware of their condition, leading to better compliance and higher QoL. While this is a plausible hypothesis, it would benefit from additional evidence. Studies suggesting that fear of invasive treatments may drive compliance in severe cases could further support this point.

**Response:** Thank you for pointing out the additional evidence to discuss the result. We agree that the fear of invasive treatments may drive compliance with the conservation treatment. However, despite an extensive literature search, we were unable to find studies specifically addressing that fear of invasive treatments drives better compliance in severe scoliosis cases. In light of this, we have revised the discussion section to clearly state the hypothesis as a potential explanation that warrants further investigation. We acknowledge the limitation of the current evidence and suggest that future research could explore this relationship in more detail.

**Comment:** Lastly, the manuscript could delve into the impact of curve patterns on gender differences. Understanding how specific scoliosis patterns affect QoL in males and females differently could provide valuable insights.

**Response:** Thanks for your insightful comment. We agree that analyzing the impact of curve patterns on gender differences could provide valuable insights into patients' quality of life. However, the sample size in each subgroup in both genders would become very small if we were to stratify by curve pattern, limiting the statistical power of the analysis. We plan to incorporate this classification in future research to further explore its effects on males and females when a larger sample size is collected. We appreciate your suggestion, which will undoubtedly guide our subsequent studies.

Conclusion
**Comment:** The manuscript effectively tackles an important topic, particularly the psychological aspects of scoliosis and their gender-specific impacts. However, incorporating more evidence, clarifying methodological choices, and revising the discussion section majorly would significantly enhance its quality and impact.

**Response:** Thank you for your positive and constructive feedback. We have revised the manuscript to include additional supporting evidence, clarified our methodological choices, and substantially improved the discussion section.

6. PLOS authors have the option to publish the peer review history of their article ([what does this mean?](https://track.editorialmanager.com/CL0/https:%2F%2Fjournals.plos.org%2Fplosone%2Fs%2Feditorial-and-peer-review-process%23loc-peer-review-history/1/010f01942ec1b508-71142361-dd96-4c73-a5a7-8e09e8c54d8f-000000/5ZxCUP999ddV6HHnTh_Th2AqeefbvaMWX3kLYau8Mq4=192)). If published, this will include your full peer review and any attached files.

If you choose “no”, your identity will remain anonymous but your review may still be made public.

**Do you want your identity to be public for this peer review?** For information about this choice, including consent withdrawal, please see our [Privacy Policy](https://track.editorialmanager.com/CL0/https:%2F%2Fwww.plos.org%2Fprivacy-policy/1/010f01942ec1b508-71142361-dd96-4c73-a5a7-8e09e8c54d8f-000000/nHVGOD-qaycbzy2N2LKBFB41S7AiSU4TLHRKlLvPoVU=192).

Reviewer #1: **Yes:** Shahrbanoo Bidari

Reviewer #2: **Yes:** Yalda Khoshhal

1.	Cella DF. Quality of life: concepts and definition. Journal of pain and symptom management. 1994;9(3):186-92.

2.	Felce D. Defining and applying the concept of quality of life. Journal of Intellectual Disability Research. 1997;41(2):126-35.

3.	Cai T, Verze P, Bjerklund Johansen TE. The Quality of Life Definition: Where Are We Going? Uro. 2021;1(1):14-22. doi: 10.3390/uro1010003.

4.	Diarbakerli E, Grauers A, Danielsson A, Abbott A, Gerdhem P. Quality of Life in Males and Females With Idiopathic Scoliosis. Spine (Phila Pa 1976). 2019;44(6):404-10. doi: 10.1097/BRS.0000000000002857. PubMed PMID: 30180148.

5.	Yagci G, Karatel M, Yakut Y. Body Awareness and its Relation to Quality of Life in Individuals with Idiopathic Scoliosis. Percept Mot Skills. 2020;127(5):841-57. Epub 20200518. doi: 10.1177/0031512520924945. PubMed PMID: 32423304.

6.	Pezham H, Babaee T, Bagheripour B, Asgari M, Jiryaei Z, Vahab Kashani R, et al. Stress level and quality of life of adolescents with idiopathic scoliosis during brace treatment. Turk J Phys Med Rehabil. 2022;68(2):231-7. Epub 20220601. doi: 10.5606/tftrd.2022.8467. PubMed PMID: 35989970; PubMed Central PMCID: PMCPMC9366478.

7.	Rivett L, Rothberg A, Stewart A, Berkowitz R. The relationship between quality of life and compliance to a brace protocol in adolescents with idiopathic scoliosis: a comparative study. BMC Musculoskelet Disord. 2009;10:5. Epub 20090114. doi: 10.1186/1471-2474-10-5. PubMed PMID: 19144157; PubMed Central PMCID: PMCPMC2635344.

8.	Misterska E, Glowacki J, Kołban M. Does rigid spinal orthosis carry more psychosocial implications than the flexible brace in AIS patients? A cross-sectional study. Journal of Back and Musculoskeletal Rehabilitation. 2019;32(1):101-9.

9.	Lin T, Meng Y, Ji Z, Jiang H, Shao W, Gao R, Zhou X. Extent of Depression in Juvenile and Adolescent Patients with Idiopathic Scoliosis During Treatment with Braces. World Neurosurg. 2019;126:e27-e32. Epub 20190129. doi: 10.1016/j.wneu.2019.01.095. PubMed PMID: 30703588.

10.	Misterska E, Glowacki M, Latuszewska J, Adamczyk K. Perception of stress level, trunk appearance, body function and mental health in females with adolescent idiopathic scoliosis treated conservatively: a longitudinal analysis. Qual Life Res. 2013;22(7):1633-45. Epub 20121128. doi: 10.1007/s11136-012-0316-2. PubMed PMID: 23188133; PubMed Central PMCID: PMCPMC3764314.

11.	Gornitzky AL, England P, Kiani SN, Yellin JL, Flynn JM. Why Don't Adolescents Wear Their Brace? A Prospective Study Investigating Psychosocial Characteristics That Predict Scoliosis Brace Wear. J Pediatr Orthop. 2023;43(1):51-60. Epub 20221005. doi: 10.1097/BPO.0000000000002272. PubMed PMID: 36194756.

12.	Karol LA, Virostek D, Felton K, Wheeler L. Effect of Compliance Counseling on Brace Use and Success in Patients with Adolescent Idiopathic Scoliosis. J Bone Joint Surg Am. 2016;98(1):9-14. doi: 10.2106/JBJS.O.00359. PubMed PMID: 26738898.

13.	Sapountzi-Krepia DS, Valavanis J, Panteleakis GP, Zangana DT, Vlachojiannis PC, Sapkas GS. Perceptions of body image, happiness and satisfaction in adolescents wearing a Boston brace for scoliosis treatment. J Adv Nurs. 2001;35(5):683-90. doi: 10.1046/j.1365-2648.2001.01900.x. PubMed PMID: 11529970.

14.	Savvides P, Gerdhem P, Grauers A, Danielsson A, Diarbakerli E. Self-Experienced Trunk Appearance in Individuals With and Without Idiopathic Scoliosis. Spine (Phila Pa 1976). 2020;45(8):522-7. doi: 10.1097/BRS.0000000000003308. PubMed PMID: 31703053.

15.	Negrini S, Donzelli S, Aulisa AG, Czaprowski D, Schreiber S, de Mauroy JC, et al. 2016 SOSORT guidelines: Orthopaedic and rehabilitation treatment of idiopathic scoliosis during growth. Scoliosis and Spinal Disorders. 2018;13(1). doi: 10.1186/s13013-017-0145-8.

16.	Linden GS, Emans JB, Karlin LI, O'Neill NP, Williams KA, Hresko MT. Early Adherence to Prescribed Brace Wear for Adolescent Idiopathic Scoliosis Is Associated With Future Brace Wear. Spine (Phila Pa 1976). 2023;48(1):8-14. Epub 20220802. doi: 10.1097/BRS.0000000000004446. PubMed PMID: 35917288.

17.	Chan S, Wong W, Kmc KC, Luk K, Wong MS, editors. The Chinese validation of trunk appearance perception scale. International Nursing Conference; 2012.

18.	Cheung KM, Senkoylu A, Alanay A, Genc Y, Lau S, Luk KD. Reliability and concurrent validity of the adapted Chinese version of Scoliosis Research Society-22 (SRS-22) questionnaire. Spine. 2007;32(10):1141-5.

19.	Vasiliadis E, Grivas TB, Gkoltsiou K. Development and preliminary validation of Brace Questionnaire (BrQ): a new instrument for measuring quality of life of brace treated scoliotics. Scoliosis. 2006;1:7. Epub 20060520. doi: 10.1186/1748-7161-1-7. PubMed PMID: 16759366; PubMed Central PMCID: PMCPMC1481574.

20.	Chan S, Cheung K, Cheung J, Chan L, Lo E, Wong M. Validation of Chinese version of brace questionnaire from its original Greek version. Journal of spine. 2019:1-6.

21.	Glattes RC, Burton DC, Lai SM, Frasier E, Asher MA. The reliability and concurrent validity of the Scoliosis Research Society-22r patient questionnaire compared with the Child Health Questionnaire-CF87 patient questionnaire for adolescent spinal deformity. Spine. 2007;32(16):1778-84.

22.	Asher MA, Lai SM, Glattes RC, Burton DC, Alanay A, Bago J. Refinement of the SRS-22 health-related quality of life questionnaire function domain. Spine. 2006;31(5):593-7.

23.	Chua YL, Toh AJN, Tan XYB, Pan DCY, Lee NKL, Lim KBL. Aspects of Patient Experience Associated With Improved Scoliosis Research Society-22 Revised (SRS-22R) and European Quality of Life Five-Dimension Five-Level (EQ-5D-5L) Scores in Patients With Adolescent Idiopathic Scoliosis Managed With Observation or Bracing. Spine (Phila Pa 1976). 2023;48(9):617-24. Epub 20230124. doi: 10.1097/BRS.0000000000004585. PubMed PMID: 36716381.

24.	Bago J, Sanchez-Raya J, Perez-Grueso FJS, Climent JM. The Trunk Appearance Perception Scale (TAPS): a new tool to evaluate subjective impression of trunk deformity in patients with idiopathic scoliosis. Scoliosis. 2010;5:1-9.

25.	Sanders JO, Newton PO, Browne RH, Katz DE, Birch JG, Herring JA. Bracing for idiopathic scoliosis: how many patients require treatment to prevent one surgery? J Bone Joint Surg Am. 2014;96(8):649-53. doi: 10.2106/JBJS.M.00290. PubMed PMID: 24740661.

26.	Li M, Wong MS, Luk KD, Wong KW, Cheung KM. Time-dependent response of scoliotic curvature to orthotic intervention: when should a radiograph be obtained after putting on or taking off a spinal orthosis? Spine (Phila Pa 1976). 2014;39(17):1408-16. doi: 10.1097/BRS.0000000000000423. PubMed PMID: 24859575.

27.	Ma Z-W, Zeng W-N, Ye K-Y. Gender differences in Chinese adolescents' subjective well-being: the mediating role of self-efficacy. Psychological reports. 2015;116(1):311-21.
