# Supplementary material for: Gender differences in trunk appearance perception and health-related quality of life (HRQoL) in the patients with moderate adolescent idiopathic scoliosis (AIS) undergoing orthotic treatment: An observational study
Source: PLoS One. 2025 Jun 25;20(6):e0325383. doi: 10.1371/journal.pone.0325383 (PMC12193679; doi:10.1371/journal.pone.0325383)
Supplement: Attachment — Submitted filename: Second Review.docx [file pone.0325383.s006.docx]

**Comments to the Author**

1. If the authors have adequately addressed your comments raised in a previous round of review and you feel that this manuscript is now acceptable for publication, you may indicate that here to bypass the “Comments to the Author” section, enter your conflict of interest statement in the “Confidential to Editor” section, and submit your "Accept" recommendation.

Reviewer #1: All comments have been addressed

Reviewer #2: (No Response)

2. Is the manuscript technically sound, and do the data support the conclusions?

The manuscript must describe a technically sound piece of scientific research with data that supports the conclusions. Experiments must have been conducted rigorously, with appropriate controls, replication, and sample sizes. The conclusions must be drawn appropriately based on the data presented.

Reviewer #1: Yes

Reviewer #2: Yes

3. Has the statistical analysis been performed appropriately and rigorously?

Reviewer #1: Yes

Reviewer #2: Yes

4. Have the authors made all data underlying the findings in their manuscript fully available?

The [PLOS Data policy](http://track.editorialmanager.com/CL0/http:%2F%2Fwww.plosone.org%2Fstatic%2Fpolicies.action%23sharing/1/010f019588b712a6-04905ecb-ece4-4e5c-be64-47023bc594b4-000000/hUtmwP-FC8Urbkq0iNS6wX5pPJA6i3-T9BKF7nPhXFc=202) requires authors to make all data underlying the findings described in their manuscript fully available without restriction, with rare exception (please refer to the Data Availability Statement in the manuscript PDF file). The data should be provided as part of the manuscript or its supporting information, or deposited to a public repository. For example, in addition to summary statistics, the data points behind means, medians and variance measures should be available. If there are restrictions on publicly sharing data—e.g. participant privacy or use of data from a third party—those must be specified.

Reviewer #1: Yes

Reviewer #2: Yes

5. Is the manuscript presented in an intelligible fashion and written in standard English?

PLOS ONE does not copyedit accepted manuscripts, so the language in submitted articles must be clear, correct, and unambiguous. Any typographical or grammatical errors should be corrected at revision, so please note any specific errors here.

Reviewer #1: Yes

Reviewer #2: Yes

6. Review Comments to the Author

Please use the space provided to explain your answers to the questions above. You may also include additional comments for the author, including concerns about dual publication, research ethics, or publication ethics. (Please upload your review as an attachment if it exceeds 20,000 characters)

**Reviewer #1:** The revisions were addressed. I think the manuscript can be published after the final confirmation from the journal.

**Response:** Thank you for confirming the revisions. We appreciate the opportunity to improve our manuscript.

**Reviewer #2:** The Authors have addressed the majority of comments from the first review which have indeed resulted in a more comprehensive and clear manuscript but unfortunately some critical questions remain unanswered. The structure of the Introduction has undoubtedly been improved and the importance of the topic is effectively articulated, nonetheless the most important matter in this section is still unresolved. It is clear that the authors have tried to demonstrate that both genders are sensitive to the issue but the initial bias about the higher sensitivity of females is still remarkably evident. This matter should either be with more literature supported or better discussed.

**Response:** Thank you for the constructive feedback. We have adjusted the introduction section in response.

**Comment:** The methodology section raised many questions in the first review, and the majority of them are, sadly, still unanswered. Validating the chosen timeline for assessments with previous studies is, though, a positive approach, however, the choice of supporting evidence is not robust.

**Response:** Thank you for your comments. We further explained the reason of the decision of the evaluation timeline in the method section, lines 150 – 161:

“The evaluation timeline was based on clinical practice and some previous studies . The first evaluation, conducted before orthosis fitting, employed only the TAPS to capture baseline trunk appearance perception [33, 34]. One month after treatment initiation, an integrated questionnaire (TAPS, SRS-22r, and BrQ) was administered to assess early adaptation. This one-month follow-up was strategically chosen because the initial orthotic treatment period is critical for long-term outcomes [35], while also aligning with standard clinical protocols that allow sufficient time for patient adaptation yet enable early orthosis adjustments if needed. The third evaluation was originally scheduled for six months after the second assessment (i.e., seven months post-orthosis fitting) to capture longer-term treatment effects, in accordance with both the 2018 SOSORT guidelines recommending 3 – 6 months follow-ups [36] and protocols from previous studies using 6-month intervals [17, 33].”

**Comment:** Furthermore, the minimum of 8-hour brace compliance is as before unjustified.

**Response:** Thank you for your comments. After careful consideration, we have removed the minimum requirement of 8-hour daily brace compliance from our inclusion criteria. Recruit patients with high compliance could introduce a bias, as it may indicate a more positive attitude to the orthotic treatment. We intended to capture a whole range of treatment behaviors and evaluate the dose-response relationship between wear time and QoL. This concern was also addressed in our initial review response.

**Comment:** On the other hand, neither the reason for using both SRS22 and BrQ together nor the explanation for the absence of SRS-22r at baseline has been clearly discussed.

**Response:** The SRS-22r is a standard clinical questionnaire that has been widely used in previous studies, allowing for direct comparisons of our results with the literature and followers. Additionally, since the SRS-22r questionnaire is used to assess the experience gone through during intervention, it is not administered at baseline. This was also mentioned in our initial review response.

**Comment:** Turning to the discussion and conclusion, it should be mentioned that the manuscript is more solid and more effectively aligned with the results, but sadly the findings are not adequately discussed and legitimated. Although most of the comments are considered and applied, unfortunately the lack of enough supporting evidence is still undeniable.

**Response:** Thank you for the constructive feedback. We have revised the discussion section in response.

Considering all mentioned above, despite the crucial matter and valuable findings, the paper should be better written and justified to be more thoroughly understood by the reader.

7. PLOS authors have the option to publish the peer review history of their article ([what does this mean?](https://track.editorialmanager.com/CL0/https:%2F%2Fjournals.plos.org%2Fplosone%2Fs%2Feditorial-and-peer-review-process%23loc-peer-review-history/1/010f019588b712a6-04905ecb-ece4-4e5c-be64-47023bc594b4-000000/9SUHgkU-2-cA6mWo3DO2K8Xaj3EG1n3w25-3cIti_60=202)). If published, this will include your full peer review and any attached files.


If you choose “no”, your identity will remain anonymous but your review may still be made public.


**Do you want your identity to be public for this peer review?** For information about this choice, including consent withdrawal, please see our [Privacy Policy](https://track.editorialmanager.com/CL0/https:%2F%2Fwww.plos.org%2Fprivacy-policy/1/010f019588b712a6-04905ecb-ece4-4e5c-be64-47023bc594b4-000000/gkc_xAl3yajhIhOeOfAGr58p_l7OersLP7T8eLAGmc4=202).

Reviewer #1: **Yes:** Shahrbanoo Bidari

Reviewer #2: **Yes:** Yalda Khoshhal
